# Quantification of silver nanoparticle uptake and distribution within individual human macrophages by FIB/SEM slice and view

**DOI:** 10.1186/s12951-017-0255-8

**Published:** 2017-03-21

**Authors:** Erik Guehrs, Michael Schneider, Christian M. Günther, Piet Hessing, Karen Heitz, Doreen Wittke, Ana López-Serrano Oliver, Norbert Jakubowski, Johanna Plendl, Stefan Eisebitt, Andrea Haase

**Affiliations:** 1 Institute for Optics and Atomic Physics, Technical University of Berlin, Straße des 17. Juni 135, 10623 Berlin, Germany; 2 Max-Born-Institute for Nonlinear Optics and Short Pulse Spectroscopy, Max-Born-Straße 2A, 12489 Berlin, Germany; 3 Institute of Veterinary Anatomy, Free University Berlin, Koserstr. 20, 14195 Berlin, Germany; 4 Department of Chemical and Product Safety, German Federal Institute for Risk Assessment (BfR), Max-Dohrn-Str. 8-10, 10589 Berlin, Germany; 5 Division 1.1 Inorganic Trace Analysis, Federal Institute for Materials Research and Testing (BAM), Richard-Willstätter-Str. 11, 12489 Berlin, Germany

**Keywords:** Nanoparticles, FIB/SEM slice and view, Absolute dose, Cellular internalization, Macrophage

## Abstract

**Background:**

Quantification of nanoparticle (NP) uptake in cells or tissues is very important for safety assessment. Often, electron microscopy based approaches are used for this purpose, which allow imaging at very high resolution. However, precise quantification of NP numbers in cells and tissues remains challenging. The aim of this study was to present a novel approach, that combines precise quantification of NPs in individual cells together with high resolution imaging of their intracellular distribution based on focused ion beam/ scanning electron microscopy (FIB/SEM) slice and view approaches.

**Results:**

We quantified cellular uptake of 75 nm diameter citrate stabilized silver NPs (Ag 75 Cit) into an individual human macrophage derived from monocytic THP-1 cells using a FIB/SEM slice and view approach. Cells were treated with 10 μg/ml for 24 h. We investigated a single cell and found in total 3138 ± 722 silver NPs inside this cell. Most of the silver NPs were located in large agglomerates, only a few were found in clusters of fewer than five NPs. Furthermore, we cross-checked our results by using inductively coupled plasma mass spectrometry and could confirm the FIB/SEM results.

**Conclusions:**

Our approach based on FIB/SEM slice and view is currently the only one that allows the quantification of the absolute dose of silver NPs in individual cells and at the same time to assess their intracellular distribution at high resolution. We therefore propose to use FIB/SEM slice and view to systematically analyse the cellular uptake of various NPs as a function of size, concentration and incubation time.

## Background

Nanotechnology is becoming a mainstream technology in modern product design. Due to their unique physicochemical properties, nanoparticles (NPs) are increasingly used worldwide in diverse applications ranging from composites in construction, fuel additives, nanomedicine or consumer products like cosmetics or textiles. NPs can enhance mechanical properties for instance in concrete, facilitate cleaning of surfaces in paints, enhance gas barrier capabilities in beverage packaging, or block ultraviolet radiation from human skin in sunscreens [1–4]. The increasing use of these materials demands proper risk assessment, which includes hazard as well as exposure assessment. Therefore, it is very important to quantify precisely the amounts of NPs, which are actually taken up into different tissues and into individual cells.

Several techniques are available for this purpose. Fluorescence based techniques such as flow cytometry and/or fluorescence microscopy are often applied. They allow for relative quantification of NP uptake while microscopy-based approaches allow insight into intracellular NP distribution, at least at medium resolution of 200 nm or larger. They do, however, require fluorescent-labelled NPs [5, 6]. Possible disadvantages may arise from dye leakage or from possible changes to the NP surface from fluorescence labelling, which may also alter uptake behaviour [7]. Furthermore, absolute quantification is typically not possible for several reasons. One being that uniform labelling of all NPs is typically not achieved, which results in differences in fluorescence yield from one NP to another. Furthermore, the determination of absolute labelling efficacy of individual particles is difficult.

A standard technique to quantify uptake of NPs is inductively coupled plasma mass spectrometry (ICP-MS). ICP-MS allows for elemental analysis and can quantify the total mass of different elements in cells or tissues. Not every element can be quantified using ICP-MS, typically this technique is applied for metals or metal oxides. Importantly, only the average dose of NPs is determined in standard measurement mode on larger cell ensembles [8, 9]. Doses per cell can be estimated by taking the total numbers of cells into account. Furthermore, standard ICP-MS does not allow to discriminate between individual NPs, agglomerates or dissolved ions. Modification of ICP-MS such as single nanoparticle ICP-MS are available that allow some quantification of individual NPs [10]. Other modifications such as laser-ablation-ICP-MS allow imaging of the sub-cellular localization of NPs with a μm-resolution [11].

In order to investigate intracellular distribution of NPs with very high spatial resolution below 1 nm, electron microscopy based techniques are required. Frequently, transmission electron microscopy (TEM) has been applied to record high resolution images of cell slices which are exposed to NPs [10, 12, 13]. In combination with spectroscopic methods (EDX), this allows the identification of the chemical composition of the particles. The major disadvantage of TEM is that only single slices and not the complete cell volume can be imaged. As a result, the absolute dose of NPs per cell is not accessible using TEM imaging. In contrast, a combination of a focused ion beam (FIB) with a scanning electron microscopy (SEM) allows the 3D imaging of NPs in a single cell, albeit at lower spatial resolution than in TEM [14–16]. This approach is known as FIB/SEM slice and view. A focused Ga^+^ ion beam is used to expose a plane cutting through the cell for SEM imaging. Consecutive thin slices of the cell are removed perpendicular to the cell substrate. An SEM image of this exposed cross-section of the cell is recorded after each slicing step [14–19]. In this fashion, one can slice through the entire cell volume. The resultant image stack can then be assembled into a 3D image of the whole cell. Sample preparation is rather simple: the cells are fixed chemically and then dried for SEM imaging [19]. The contrast of metallic NPs embedded in an organic matrix such as a cell is intrinsically high when imaging by SEM, due to the high secondary electron yield of metallic particles [20]. Thus, for metallic NPs counter-staining is not required, in contrast to e.g. the use of osmium in conventional TEM or the use of specific labels in fluorescence microscopy.

The lateral spatial image resolution of conventional SEM is about 1 nm and the depth of the consecutive FIB slices can be decreased to a few tens of nm. Thus, FIB/SEM slice and view is a suitable method to image a large number of different types of NPs in cells and to determine the absolute dose and the intracellular distribution. Hence, FIB/SEM is an ideal combination of a high resolution intracellular imaging and a precise quantification approach for intracellular dose providing single cell resolution. As for TEM, an EDX detector can add spectroscopic information to each single-slice image and, thus, to the complete model of the cell. A major drawback for widespread application of this technique for this purpose, however, is the fact that NP numbers per cell are not readily available from the images. NPs numbers per cell may be obtained by manually counting the particles, which is time-consuming and prone to artefacts in particular when many agglomerates are present. Automated evaluation, however, is in practice a prerequisite for systematic studies involving many samples. Beyond image detection and accounting for shading effects, this also involves taking the physics of contrast generation into account, as given by the escape depth of secondary electrons within an organic matrix. Commercial software applications are not available for this purpose. Therefore, we have developed a model allowing for automatic analysis of FIB/SEM images to retrieves information on the NP numbers per cell. With our approach it is now possible to determine absolute doses as a function of particle sizes, exposure times or concentrations for various types of NPs. These data are expected to give additional impact to toxicological in vitro studies.

## Results and discussion

### Characterization of silver NPs

We used citrate-coated silver NPs with a diameter of 75 nm, herein referred to as Ag 75 Cit. We have already used this type of nanosilver in a previous study to quantify cellular uptake using different ICP-MS based approaches [21]. This allows us to compare the results of both studies. NP characterization is described in detail in [21] and is summarized in Table 1.

**Table 1:** Characterization of Ag 75 Cit. Data are taken from [21]

		Dispersion in water	Dispersion in CCM	Dissolution in water (%)
	TEM (nm)	DLS (nm)	DLS (nm)	
Ag 75 Cit	74 ± 8	79 ± 0.5	71 ± 0.2	1 ± 0.03 to 3.1 ± 0.7

TEM value of Ag 75 Cit was reported by NanoComposix, Prague, Czech Republic [22]. DLS data are presented as mean and standard deviation. Dissolution was determined at 2 and 10 μg/ml, at lower concentrations higher dissolution rates were observed

In pure water, the NP diameter was determined via dynamical light scattering (DLS) to be 79 ± 0.5 nm and in cell culture medium (CCM) to be 71 ± 0.2 nm. This is in good agreement with data from TEM measurements (74 ± 8 nm) provided by the Manufacturer (NanoComposix) [22].

Furthermore, we have analysed the cell viability using the WST-1 assay in THP-1 derived macrophages that were incubated with Ag 75 Cit in concentrations up to 100 μg/ml. We did not observe any significant reduction in cell viability after 24 h. In our experiment we used a much lower concentration of 10 μg/ml Ag 75 Cit with an incubation time of 24 h, which is clearly non-toxic to the cells.

### Silver NP detection using FIB/SEM slice and view

**Fig. 1:**
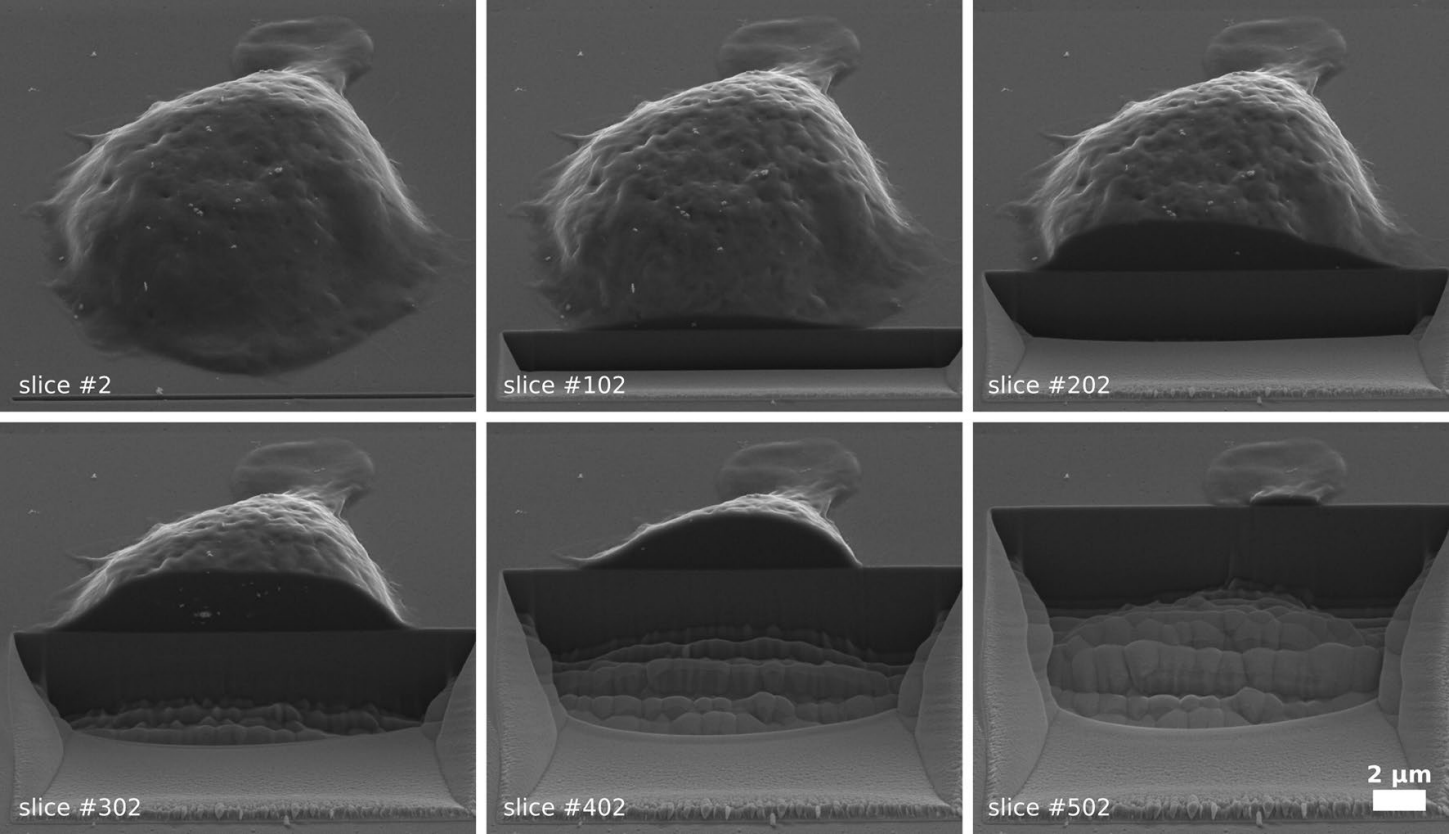
FIB/SEM slice and view. Gradual degradation of a single THP-1 macrophage using FIB/SEM slice and view. Here, six sample images of the slicing process are shown. In total, 625 images were taken to record the complete cell. A single slicing step removes 40 nm of the cell. The Ag 75 Cit NPs are visible as* bright spots* in the cell

A single macrophage cell was imaged by the FIB/SEM slice and view technique. In each slicing step 40 nm of the cell was removed and the cross section was imaged by SEM. The 40 nm slice separation ensured that each silver NP was cut at least once in the process. In total 625 images of the cell were recorded. Six slices of this cell are shown in Fig. 1.

During the chemical fixation process of the sample the structure of the intracellular cell compartments is not preserved. As a result, the inner part of the cell is visible as a homogeneous grey area in the SEM images and the Ag 75 Cit NPs can be readily identified within the cells as bright spots within a background of this cell matrix. In this situation the NPs show up bright as the secondary electron yield of silver is approximately twice as high as that of organic carbon [20, 23]. Note, that Carbon NPs do not show such a high contrast as the secondary electron yields of an organic matrix and Carbon are similar to each other.

Both, the homogeneous intensity distribution of the cross-section of the cell and the high contrast of the silver NPs within the cell simplifies the detection of the cell shape and of the incorporated silver NPs during the segmentation process. The shape of the cell and the silver NPs are detected using threshold and edge detection algorithms. Details about the procedure can be found in the methods section.

**Fig. 2:**
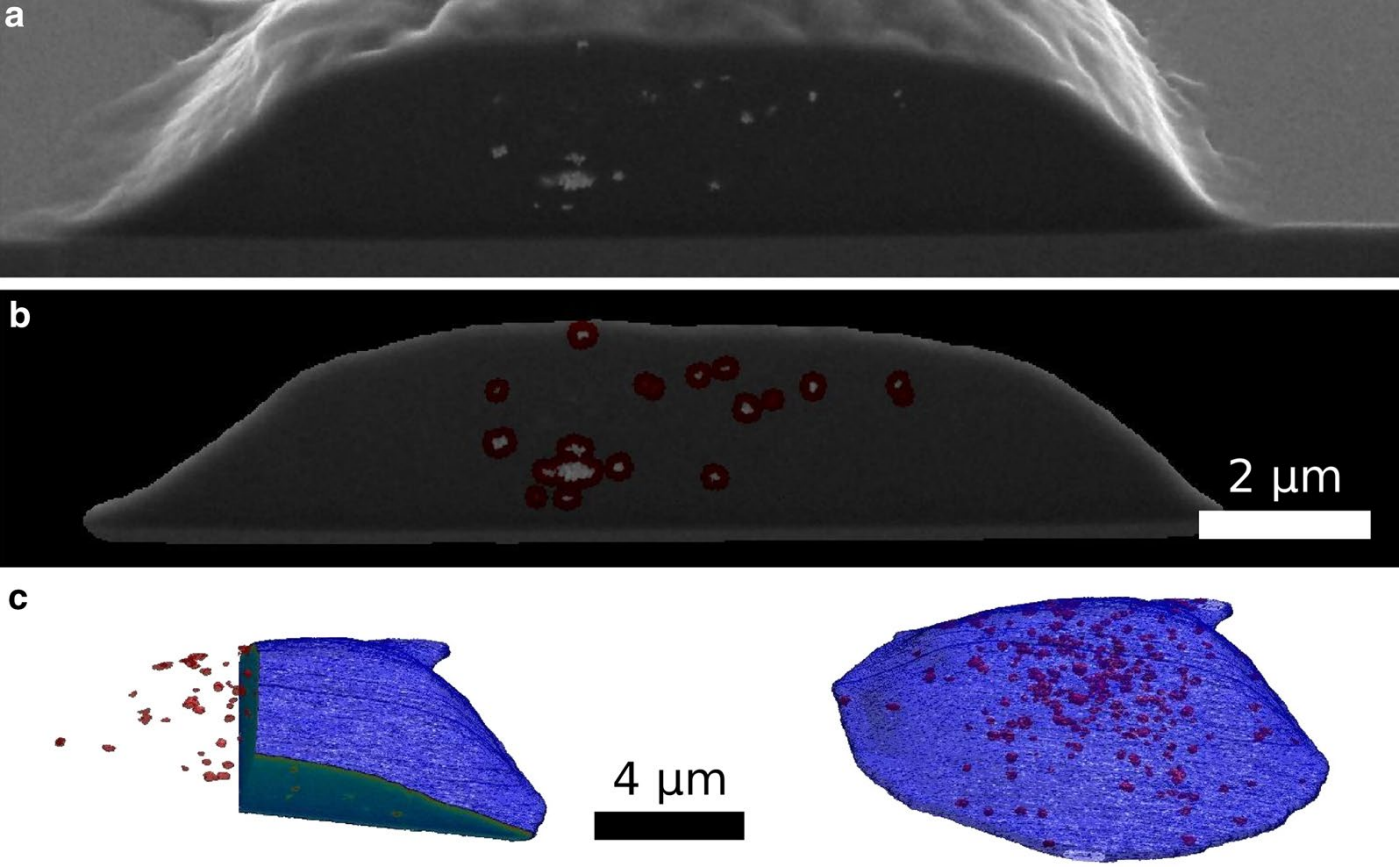
Cell and silver NP segmentation. In** a** one single slice of the cell (slice 238) is shown before segmentation. In** b** the segmented shape of the cell as well as the detected Ag 75 Cit NPs (highlighted in* red*) are presented. Based on the segmentation, a 3D model (**c**) of the cell (*blue*) and the silver NPs (*red*) is calculated. In the* left panel*, two orthogonal cross-sections through the cell are shown

In Fig. 2 a sample image of cell is shown before (a) and after segmentation (b). To ensure that the complete cell is segmented, the shape of the segmented cell is slightly increased. This region appears as a light grey area around the cell. The segmentation process comes to a limit at cross-sections where the cell height above the substrate is very low. This is the case for the rear part of the cell, which can be seen in Fig. 1 at slice 502. Here, the contrast of the inner cell matrix is not high enough for thresholding and these areas of the cell are not segmented. As no silver NPs are located in this very small remaining part of the cell as verified by visual inspection of the SEM images of the slices, we decided not to adjust the algorithm.

In a second step, we segmented the silver NPs within the cell (Fig. 2b). The segmentation process is applied to each NP cluster individually. In this way, intensity fluctuations of the SEM signal generated by the silver NPs relative to the cell matrix can be compensated.

Based on this segmentation a 3D surface model of the cell as well as of the silver NPs is calculated. This is depicted in Fig. 2c.

### Characterization of single silver NPs

In the simplest model, the total number of silver NPs could be calculated from the segmentation data, if the number of voxels corresponding to a single NP is known and then used for calibration. In principle, such calibration data can be extracted from the present data set, as it contains several isolated NPs. However, in order to determine the absolute dose of silver NPs within the cell, the image formation process of SEM imaging needs to be taken into account to avoid quantification artefacts from this simple model. Such artefacts come about as silver NPs are not only visible if they are exposed in the surface layer defined by the respective cross-section slice, but can already be detected in the SEM if they are located below the surface. This is due to the fact that the escape depth of secondary electrons can be larger than the slicing interval of 40 nm in our case [24]. As a result, the silver NPs appear larger than they actually are.

**Fig. 3:**
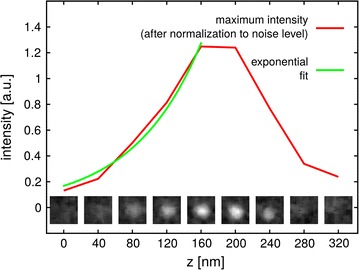
Analysis of a single silver NP. Nine slices in the immediate vicinity of a single silver NP at a distance of 40 nm to each other are shown above the* x-axis*. The maximum intensity of each image is taken to calculate the escape depth of electrons. The escape depth for the particle shown is ~79 nm. The average escape depth for all 16 single silver NPs investigated in this fashion is 89 ± 17 nm. The escape depth is used to determine total normalized intensity of a segmented single SNP in our sample to be (89 ± 17)

To quantify this effect, the escape depth of electrons in the cell matrix has to be determined. In a second step, this information can be used to correct the apparent volume of a single silver NP. For quantification, 16 single silver NPs were selected by hand and then analysed. The average escape depth is determined from consecutive slices as shown in Fig. 3 and amounts to 89 ± 17 nm. Without this correction the total voxel size for a single silver NP would be 230 ± 69 voxels (each voxel has a size of 6.3 nm × 6.3 nm × 40 nm). However, after correction the voxel size was determined to be 134 ± 23 voxels, which corresponds to ~210,000 ± 36,000 nm^3^ and thus, fits very well to the volume of a 74 nm silver NP (calculated to be 212,000 nm^3^). We note that this correction is significant in order to obtain reliable quantifications. From our data, the average projected NP size can also be extracted and was calculated to be 109 ± 16 pixel. This value is equivalent to a particle diameter of 74 ± 10 nm and in good agreement with the values of the manufacturer in Table 2.

**Table 2:** Size determination of the silver NPs from the SEM images

	Experimental data	Calculated using manufacturer’s data [22]
Escape depth of electrons within the cell matrix (nm)	89 ± 17	
Volume of a single silver NP (nm^3^)	210,000 ± 36,000	212,000 ± 2400
Diameter of a single silver NP (nm)	74 ± 10	74 ± 8

We conclude that the parameters of a single Ag 75 Cit NP extracted from the SEM images are in close agreement with the values provided by the manufacturer and complementary characterization. Furthermore, this result also proves that our algorithm are able to segment the silver NPs within the cell. The results are summarized in Table 2.

### Characterization of single silver NPs

To analyse all segmented silver NPs within a single cell and thus to get the absolute internal dose, the voxel size of each cluster (which is proportional to its volume) needs to be calculated taking into account the escape depth of the electrons for correction.

**Fig. 4:**
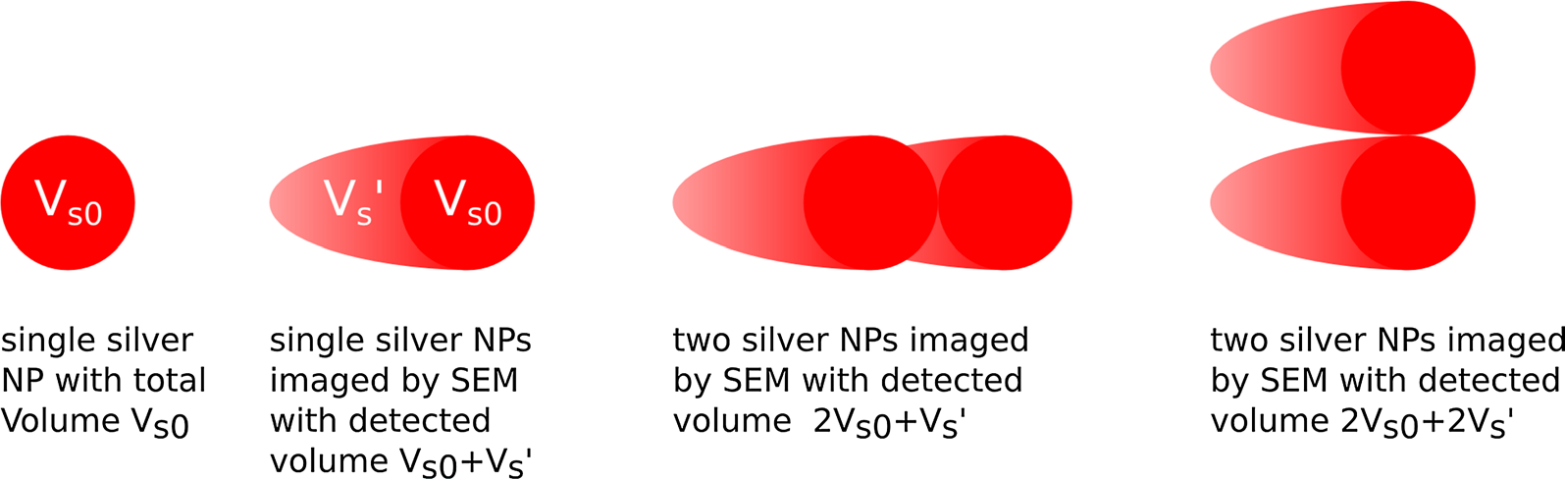
Simple model of the imaging process of silver NPs by SEM. Due to the escape depth of the electrons within the cell matrix, the detected silver NP volume appears elongated along the optical axis of the SEM. To correct for this effect, the escape depth of the electrons and the shape of the NP clusters needs to be taken into account

Before performing this analysis, two cases depicted in Fig. 4 have to be investigated in more detail. After slicing, when two adjacent silver NPs are imaged with the SEM and when they are located next to each other within the slicing plane, they are imaged like two individual NPs (see Fig. 4). The situation is different, if the silver NPs are located in different depth along SEM optical axis, specifically, if they are located behind each other in different slices within the electron escape depth. As the escape depth of electrons within the NPs is much smaller than the escape within the cell matrix, all electrons escaping from the posterior NP (further away from the slice surface) are absorbed by the anterior NP (closer to or at the surface). Thus, the posterior NP will only be detected after the anterior NP is removed by the FIB. As a result, the apparent elongation due to the escape depth has to be considered only for one silver NP to calculate the correct voxel size of this kind of cluster (see Fig. 4).

Therefore, to determine the corrected voxel size for larger clusters, the projected area of the cluster with respect to the cross-section plane needs to be calculated. Then the escape depth can be used for correction and thus the volume of each cluster can be calculated. Details about this procedure can be found in the methods section.

**Fig. 5:**
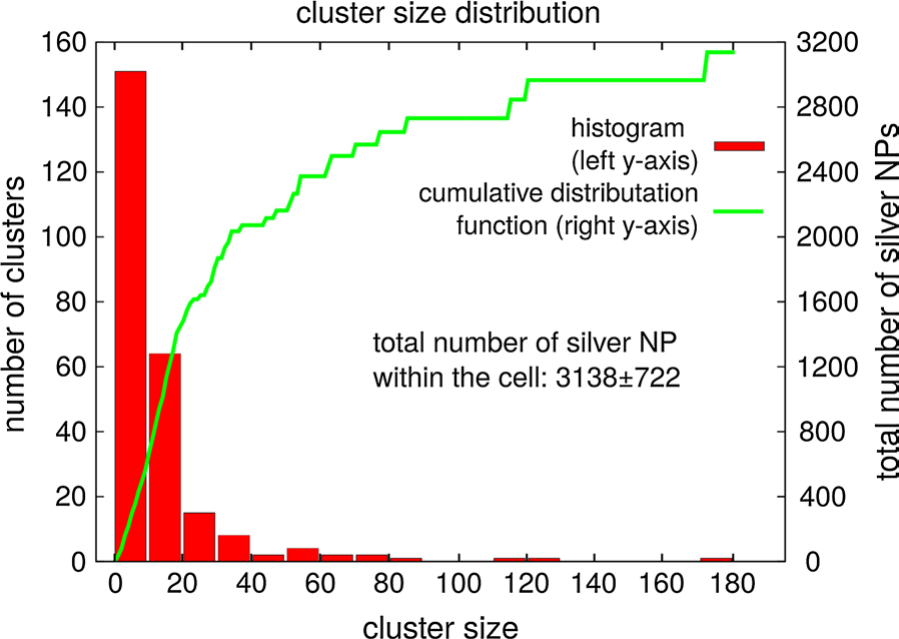
Cluster size distribution of uptaken silver NPs. Histogram and cumulative distribution function of all silver NPs within the cell. The absolute dose of silver NPs within the cell was 3138 ± 722. Although only a few clusters with a size larger than 20 NPs were present in the cell, most NPs were located in these larger clusters (~53%). Only 9% of all silver NPs were found in very small clusters (cluster size 1–5, corresponding histogram not shown). The binning width for the histogram is 10 silver NPs. The* left y-axis* corresponds to the histogram and the* right y-axis* to the cumulative distribution function

After taking this correction into consideration a total of 3138 ± 722 Ag 75 Cit NPs were detected within this single macrophage cell. Most of the NPs were found in large agglomerates. 53% of all NP were located in clusters with a size of at least 20 NPs. Only 9% of all silver NPs were found in clusters of one to five particles. Only 34 single silver NPs were detected within the single macrophage (Fig. 5).

**Fig. 6:**
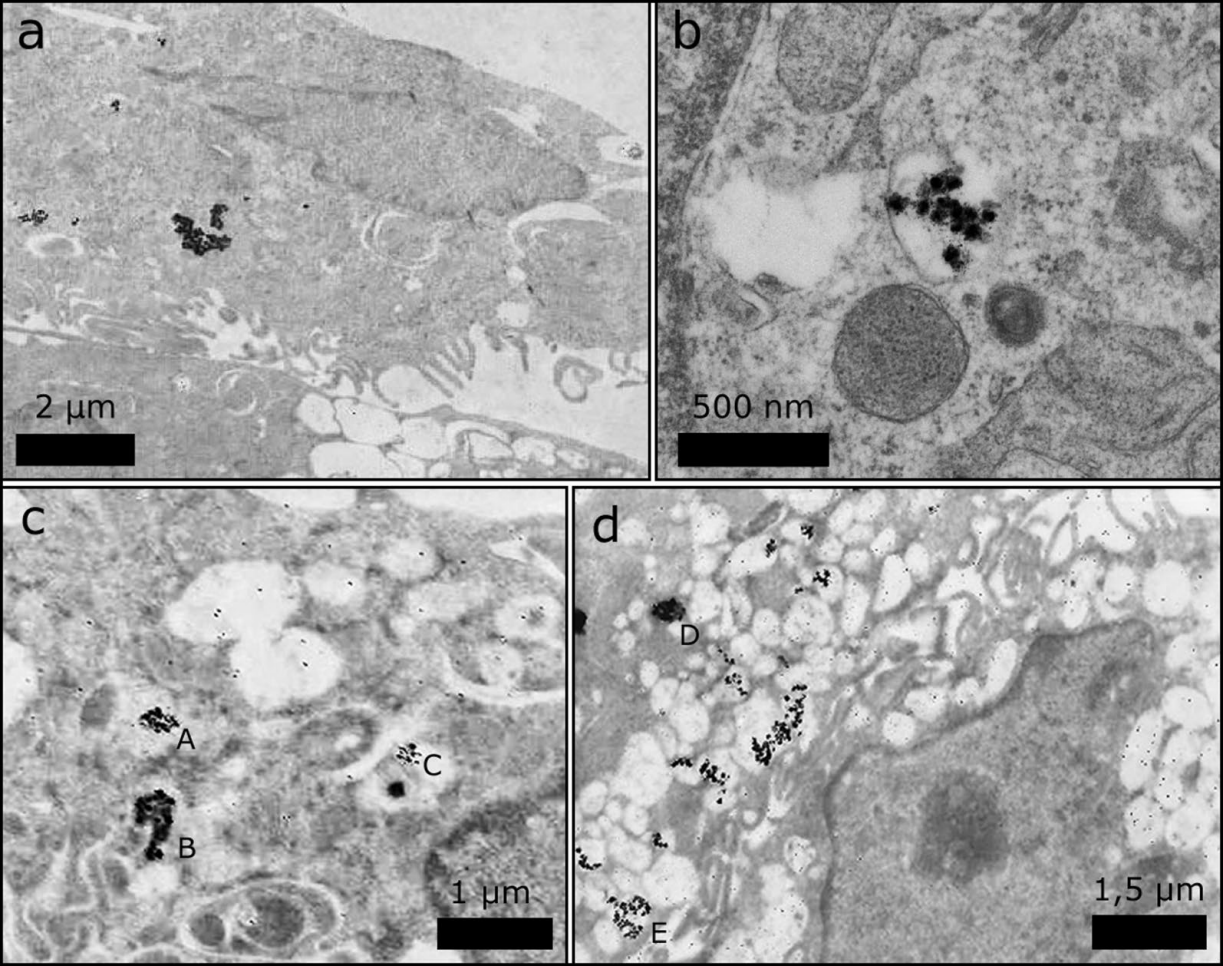
TEM images of slices through THP-1 cells with uptaken silver NPs. In the TEM images, the silver NPs appear as* dark spots* in **a**–**d**. From all images it can be seen that most silver NPs are found in loosely- or densely-packed agglomerates. The larger magnification** b** reveals that silver NPs are located inside membrane-enclosed structures, which likely represent phagolysosomes. In** c** and** d** exemplary sizes of several agglomerates were determined. The agglomerate sizes are* A* = 336 nm;* B* = 344 nm;* C* = 224 nm;* D* = 425 nm;* E* = 525 nm

Most of the silver NPs are found in large agglomerates. This can be caused by (a) the formation of agglomerates outside the cell, which are then taken up by phagocytosis, or (b) by the uptake of single silver NPs or small agglomerates that once inside the cell create larger clusters, e.g. after fusion of endocytotic vesicles to phagolysosomes. From our FIB/SEM data alone, this cannot be clarified. In addition, our data do not allow to discriminate whether the silver NPs are located inside organelles or are located within the cytoplasm. To clarify this point, we performed a TEM analysis of cells grown in the same culture and found that the silver NPs are always detected inside membrane-enclosed structures, which most likely are phagolysosomes (Fig. 6).

Others studies confirm that NPs are located inside membrane-bound structures in macrophage cells [25–27]. However, FIB/SEM slice and view may also be applied to unravel more details on uptake mechanisms. To this end it would be necessary to improve the sample preparation protocol such that the cell organelles are preserved and to analyse cells after several different incubation times to follow and quantify cells being present in different organelles over time.

### Quantification of silver NP uptake using ICP-MS

To further cross-check our results, we also determined the amount of silver NPs taken up by using the well established ICP-MS approach with the same experimental treatment conditions. In total three and six different replicates for the control and the samples incubated with silver NPs respectively were measured by ICP-MS. We found 2613 ± 271 silver NPs within the cells. This coincides very nicely with the results obtained using FIB/SEM slice and view and therefore validates our new approach to determine the absolute dose in single cells.

Our result is also in good agreement with other studies. For the same type of silver NPs also treating the cells (Neuro-2a cells) with a silver NP concentration of 10 μg/ml for 24 h, Hsiao et al. [21] determined the absolute dose of silver NPs as 1474–1740 silver NPs by SP-IPC-MS. They used the same type of silver NPs and also treated the cells with a silver NP concentration of 10 μg/ml for 24 h. The difference to our results can be explained as follows. Firstly, Hsiao et al. used a neural cell type, Neuro-2a cells. It may be expected that macrophages accumulate more silver NPs than other cell types, given their biological function is to incorporate foreign particles and to remove them. Secondly, when when Hsiao et al. determined the absolute dose by complementary analysis using laser ablation ICP-MS, they find that the cell-to-cell variation of silver NPs uptake is very high. In contrast, we analyzed only a single cell.

We show that FIB/SEM slice and view can be applied to quantify silver NPs within a single cell as well as to image silver NP distribution inside the fixed cell. As the silver NPs are imaged directly by SEM, the number of silver NPs per agglomerate and thus the absolute dose can be determined. Inclusion of the effects of the electron escape depth as well as shading of particles were found to be crucial for quantitative and automatable analysis. To our knowledge, this is the first time that the absolute dose of silver NPs could be determined within a single cell, i.e. without resorting to averaging over a large cell ensemble. Here, we found that 3138 ± 722 silver NPs were located inside the cell investigated. The cluster size distribution was determined and most of the NPs were found to be agglomerated in clusters with a size larger than 20 NPs.

## Conclusion

As a proof-of-principle experiment we determined the absolute dose of uptaken silver NPs in a single cell by FIB/SEM slice and view. This method can be used to investigate other types of NPs that are readily detected by SEM provided that the interslice distance of the FIB is smaller than the NP diameter. In fact, even NPs somewhat smaller than the slice spacing should be detectable taking the electron escape depth into account. We see potential for our approach to analyse the uptake mechanisms of NPs as a function of size, concentration and incubation time. Without having to resort to averaging over many cells, the variance of these processes in larger cell ensembles could be investigated. Furthermore, the approach presented here can be used as a complementary method for toxicological studies and as a calibration tool for methods that can only determine the *relative* dose of NPs in cells or observe other parameters depending on NP uptake and toxicity.

## Methods

### Nanoparticles

Commercially available 75 nm Ag nanospheres (Biopure quality), carrying a citrate modification, were used (NanoComposix, Prague, Czech Republic) [22]. The stock solution of silver NPs was first ultrasonicated for 5 min. Then the NPs were diluted directly into the indicated cell culture medium (see below) at the desired final concentration and applied to the cells. NP suspensions were freshly prepared for each experiment.

### Characterization of nanoparticles

The hydrodynamic size of the Ag 75 Cit NPs in water and in cell culture medium was monitored using a Zetasizer Nano ZS apparatus (Malvern Instruments GmbH, Herrenberg, Germany).

### Cell culture

THP-1 cells (ACC 16 from DSMZ, Braunschweig, Germany) were cultured in RPMI 1640 medium supplemented with 10% fetal calf serum (FCS), 1% L-glutamine, 1% penicillin/streptomycin, 1% Hepes and 1  sodium pyruvate. Phorbol-12-myristate-13-acetate (PMA) at 100 ng/ml for 24 h was used to differentiate THP-1 cells into macrophages. Cells were cultivated at 37 °C, 5% CO_2_ and 95% relative humidity on silicon wafers from crystec (Berlin, Germany).

### Cytotoxicity

The WST-1 cell viability assay was used to evaluate the toxicity of Ag 75 Cit NPs according to manufacturer instructions (Roche Diagnostics; Mannheim, Germany). Cells were treated 24 h after seeding in 96-well plates and had been treated with 5, 10, 20, 30, 50 and 100 μg/ml silver NP for 24 h using three technical replicates per dose. As a positive control known to to be toxic to the cells, 10 μl DMSO was used. Interfering NP and cells were removed in a table top centrifuge by centrifugation with maximum speed prior to spectrophotometric read-out. Supernatants were analyzed using a plate reader (TECAN, Switzerland) at 450 nm. The experiment was performed using three independent biological repeats.

### Cell incubation and sample preparation

For analysis using FIB/SEM slice and view, the cells were exposed to 10 μg/ml Ag 75 Cit NPs for 24 h. This dose has been selected based on comparisons to doses used in in vivo inhalation studies. In inhalation studies cellular overload corresponds to a cellular dose of approximately 90–120 pg NP per macrophage cell [28, 29]. Therefore, the dose chosen here is a realistic dose likely to occur during in vivo inhalation studies but should be well below overload conditions und is clearly a non-toxic dose. Cells were washed three times with DPBS before being fixed with paraformaldehyde (4% in DPBS, Carl Roth GmbH, Karlsruhe, Germany). The citrate coating of the silver NPs make sure that their position is preserved while chemical fixation. After that the cells were washed again three times with DPBS. The medium was replaced by serial dilution with acetone (Carl Roth, Karlsruhe, Germany): 30% acetone, 50% acetone, 70% acetone, 90% acetone, two times 95% acetone and three times 100% acetone. Finally, the cells were dried using critical point drying.

### ICP-MS analysis

An ICP-MS Sector Field instrument, ICP-SFMS (Element XR, Thermo Fisher Scientific GmbH, Bremen Germany) equipped with a concentric nebulizer (Micromist 0.2 ml, L90350, AHF) and a conical spray chamber (ML145026, Meinhard) with an impact bead was used for the experiment. The ICP-MS instrumental and operational parameters are given in Table 3. Before analysis, the ICP-MS was tuned using an aqueous multi-element standard solution (1 ng/ml each of Li, In and U) for consistent sensitivity and minimum levels of doubly charged ions and oxide species of Ce. The time-resolved analysis (TRA) mode was used and thus intensities were collected as a function of time (counts per second). The data of this experiment were recorded using Thermo Plasma Lab software.

**Table 3:** Operating parameters for the ICP-MS element XR

Parameter	Values
Rf power	1550 W
Ar cooling gas flow rate	15 l/min
Ar auxiliary gas flow rate	1 l/min
Sample and skimmer cone	Nickel
Micronebulizer	Micromist 200 μl
Data acquisition mode	Time resolved analysis (TRA)
Isotope	Ag^107^, In^115^
Uptake rate	0.4 ml/min
Dwell time	0.1 ms
Acquisition time	65 s

Silver and Indium ICP Standard, 65% w/w ultrapure grade nitric acid and hydrogen peroxide were purchased from Merck (Darmstadt, Germany). Milli Q water from purification system Millipore gradient, (Merck MilliPore, Darmstadt, Germany) was used for dilution of concentrated nitric acid.

For ICP-MS analysis the THP-1 cells were cultivated and treated as for the FIB/SEM analysis, i.e. incubated 10 μg/ml Ag 75 Cit NPs for 24 h. The average concentration of NPs in THP-1 cells was determined by ICP-MS after cell digestion. An aliquot of the washed cells containing approximately 10^5^ cell/ml was digested overnight with 0.15 ml concentrated nitric acid and 0.05 ml hydrogen peroxide at room temperature. The digested solution was freshly diluted and the total Ag content uptake per single cell was determined by calibration with dissolved silver standards. Indium was added to the digested samples as internal standard in order to correct the instrumental variations in sensitivity during the analysis.

### TEM analysis

TEM processing was performed as previously described [30]. Cells in the culture dish were washed with phosphate buffered saline (PBS) and fixed overnight by immersion with Karnovsky’s fixative at 4 °C. After three washes in 0.1 M cacodylate buffer, postfixation was performed with 2% osmium tetroxide in 0.1 M cacodylate buffer for 1 h at 4 °C. After another three washes in 0.1 M cacodylate buffer, cells were removed from the culture dish and centrifuged at 2000×*g* for 5 min. The resultant pellet was then coated with 1.5% agar (Merck Eurolab, Darmstadt, Germany) for 30 min at 4 °C. Subsequently the agar with the attached cell layer was removed from the wells. The samples were dehydrated in an ascending ethanol series (30–100% alcohol v/v) and embedded in Epon using beem capsules (Plano, Marburg, Germany). Polymerisation was carried out at 60 °C for 24 h. Semithin sections (1 μm) were cut on an Ultracut E ultramicrotome (Reichert-Jung, Vienna, Austria) with a diamond knife, stained as published elsewhere [30] and analyzed by light microscopy. Ultrathin sections (60 nm) were cut with a diamond knife, mounted on copper grids (Plano, Marburg, Germany) and examined with a Zeiss 10CR electron microscope (Jena, Germany).

### FIB/SEM slice and view

For FIB/SEM slice and view a FEI Helios NanoLab 600 system (FEI Eindhoven, Netherlands) was used. To increase the electric conductivity of the samples they were coated with platinum (about 5 nm) using ion beam induced deposition (30 kV, 146 pA). After that a single cell was sequentially sliced by the FIB (Ga^+^ ions using 30 kV, 146 pA). In each step, a slice of the cell (thickness 40 nm) was removed perpendicular to the silicon wafer surface. The remaining cell was imaged by the SEM (5 kV, detecting secondary electrons, magnification 5000×). This process was repeated until the complete cell was consumed and imaged. As SEM amplification and contrast settings remained unchanged during the stack of slice images, the intensity per pixel, and hence also per resulting voxel reflects the amount of secondary electrons generated with a constant proportionality factor throughout the sliced volume. We denote these units as "arbitrary intensity units" (a.i.u.). Integrating this intensity over space after suitable segmentation results in a measure of particle volume, which is for convenience also measured in a.i.u. Note, that the SEM images were taken under an angle of 52° to the surface normal as FIB and SEM columns are by design not collinear to each other. The resulting distortion of the image can be corrected automatically using computer algorithms.

### Data analysis: cell and silver NP segmentation

To correct for image drift during the slice and view process, cross-correlation was used to determine the shift between pairs of images. Afterwards, the distortion caused by the different working angle between FIB and SEM image was corrected by undistorting the image. Due to the preparation process the inner cell matrix was homogeneous which allowed the use of a threshold algorithms to detect the shape of the cell in each image. Before applying this algorithm, a Wiener filter was used to reduce the noise level of the images.

The FIBing process produced a trench in the substrate at the bottom of the cell. As this trench moves along with the position of the slicing plane, it can be used to calculate the lower boundary of the cell shape. Afterwards the threshold algorithm can be applied. The upper boundary of the cell does not have sharp edges but had a smooth transition. To make sure that the complete cell is segmented from the image, the shape of the cell was extended by 10 pixels via a convolution.

When silver NPs were located close to the boundaries of the cell, the threshold algorithm produced a notch in the outer boundary as the intensity level of the silver NPs in the image was much higher than the intensity level of the cell. This was corrected by detecting the silver NPs close to the boundaries of the cell shape by thresholding and removing the notches by stepwise increasing the boundary shape of the cell shape at this position until it was almost at the same intensity level as the surrounding area. Finally, the detected boundary of the cell was smoothed by a convolution kernel to remove any remaining edges and notches.

The silver NPs within the cell were detected in a two-step approach. First, the images were low pass filtered for contrast enhancement of the edges of the silver NPs. The rough position of the silver NPs was found by applying an edge detection algorithm on the low-pass filtered images (Roberts edge detection) [31]. In the second step the shape of the silver NPs was refined individually for each cluster in 3D. For that, the mean grey level intensity of each cluster and of the surrounding cell matrix was determined using Otsu’s multi threshold method [32]. This was necessary as the intensity levels of the silver NPs and of the cell can fluctuate with respect to the position of the cluster in the cell. The determined values were then used to segment each cluster of silver NPs within the cell shape.

### Data analysis: voxel size of a single silver NP

To calculate the total number of NPs and the size of the clusters, the segmented boundary of the silver NPs were used. First, the voxel size of a single NP was analysed. To this end 16 single NPs were identified “manually” without automation. In Fig. 3 the slices through one of these silver NP are shown. In the segmentation process, this silver NP is detected in slices 3, 4 and 5. For better comparison and to minimize background effects, the intensity of the segmented silver NPs is normalized and then accumulated. The average total normalized intensity for all 16 single silver NPs is 160 ± 42 a.i.u.

As the detected electrons have a specific escape depth *l* within the cell matrix, silver NPs which are located in deeper layers of the cell will be detected before they are sliced using the FIB [20, 23, 24]. As a consequence the detected volume $V_s = (V_{s0}+V_s') = 160\pm 42$ a.i.u. of a single silver NPs appears larger than the actual volume $V_{s0}$ (see Fig. 4). Here, $V_s'$ is the offset volume caused by electrons escaping from deeper layers.

To determine the escape depth, the maximum intensity (which is normalized to the surrounding noise) of each slice is used for each of the 16 single silver NPs and an exponential function is fitted to the data points (see Fig. 3). For the silver NP shown in Fig. 3, the escape depth is $l = 79$ nm and the average escape depth for all 16 silver NPs is $l = 89\pm 17$ nm. $V_s'$ can now be calculated by multiplying $V_s$ with $\exp (-1/l \times 40$ nm) (40 nm is used as each slice has a depth of 40 nm). The result is shown in Table 4.

**Table 4:** Intensity parameter for single silver NPs

	Total intensity
Detected volume $V_s$	160 ± 42
Offset volume $V_s'$	62 ± 26
Actual volume $V_{s0}$	98 ± 18

### Data analysis: cluster analysis and total number of silver NPs

To calculate the number of silver NPs in a cluster $n_c$, the shape of the cluster needs to be considered to correct for the offset volume of the cluster $V_c'$. If the actual volume for a cluster $V_{c0}$ can be extracted from the detected volume $V_c$ for each cluster, the number of silver NPs in this cluster can be determined by $n_c = V_{c0}/V_{s0}$. Assume a cluster of two silver NPs. If the silver NPs are located next to each other within the plane of the cross-section, the detected total normalized intensity of the cluster is $V_c = V_c' + V_{c0} = 2\times (V_{s0} + V_s')$ (see Fig. 4). If both silver NPs are located behind each other in different slices, the secondary electrons emerging from the posterior NP are absorbed by the anterior NP. As a result, the detected total normalized intensity is $V_c = V_c' + V_{c0} = 2\times V_{s0}+V_s'$ (see Fig. 4).

To analyse larger clusters, the projected cluster area $A_c$ normal to the plane of the cross-section needs to be determined to get $V_c'$. From $A_c$ the number of projected silver NPs $n_p$ can be calculated by $n_p = A_c/A_s$. Here, $A_s$ is the projected area of a single silver NP which is $A_s = 109\pm 16$ pixels. This can be used to get $V_c' = n_p\times V_s'$. As a result, the total number of silver NPs within a cluster can be calculated by $n_c = (V_c - V_s'\times A_c/A_s )/V_{s0}$.

Note, that it is not necessary to correct for the angle between the normal to the plane of the cross-section and the SEM column as the escape of the secondary electrons from the cell matrix is related to the shortest path length within the matrix (which is orthogonal to the plane of the cross section). In the case of two silver NPs located above each other in different slices, it is theoretically possible, that the secondary electrons from the posterior silver NP escape by moving around the anterior silver NP through the cell matrix. But this effect can be neglected as the escape probability decays exponentially with increasing path length.

## Abbreviations

CCM: cell culture medium
CCT: collision cell technique
DLS: dynamic light scattering
DPBS: Dulbecco’s phosphate buffered saline
FCS: fetal calf serum
FIB: focused ion beam
ICP-MS: inductively coupled plasma mass spectrometry
NPs: nanoparticles
MEM: minimal eagle’s medium
SEM: scanning electron microscopy
TEM: transmission electron microscopy
